# Effect of Applying 1% Metformin on Guided Bone Regeneration Processes with Bovine-Derived Xenografts

**DOI:** 10.3390/jcm13102973

**Published:** 2024-05-18

**Authors:** Oier Montalbán-Vadillo, Esteban Pérez-Pevida, Iratxe Viteri-Agustín, David Chávarri-Prado, Alejandro Estrada-Martínez, Markel Diéguez-Pereira, Fernando Sánchez-Lasheras, Aritza Brizuela-Velasco

**Affiliations:** 1 Department of Surgery, Faculty of Medicine, University of Salamanca, 37007 Salamanca, Spain; 2 Faculty of Health Sciences, Miguel de Cervantes European University, 47012 Valladolid, Spain; 3 EDE-SRGROUP, La Salle Higher Center for University Studies, 28023 Madrid, Spain; 4 University Institute of Space Sciences and Technologies of Asturias (ICTEA), University of Oviedo, 33004 Oviedo, Spain; 5 Department of Mathematics, Faculty of Sciences, University of Oviedo, 33007 Oviedo, Spain

**Keywords:** bone regeneration, metformin, xenograft

## Abstract

**Background:** Although xenografts have shown successful results in GBR procedures due to their osteoconductive properties, many authors have opted to add co-adjuvant drugs to favor osteogenesis and differentiate cells into an osteoblastic lineage. Metformin has been shown to have bone-protective properties, regulating osteoclast differentiation, as well as the ability to promote osteoblast mineralization and differentiation. The present study aimed to evaluate the effect of the local application of a 1% metformin solution on bone neoformation in the treatment of an experimental bone defect in a guided bone regeneration animal model with a particulated bovine hydroxyapatite xenograft with hyaluronate. **Methods:** With this purpose in mind, two critical defects with 8 mm diameter and 0.5 mm depth were created in eight male New Zealand rabbit calvarias. Titanium cylinders were fixed in each defect and filled with particulate hydroxyapatite of bovine origin and sodium hyaluronate, with sterile injectable saline added to the control group and sterile 1% metformin solution added to the test group. At 6 weeks, the animals were euthanized, and samples were obtained and prepared for histomorphometric analysis. **Results:** A higher percentage of new bone formation was observed in the metformin samples than in the control samples, both in the region closest to the animal’s calvaria and in the most distal region analyzed. A higher average bone–biomaterial contact percentage was observed in the samples, with metformin in both the proximal and distal regions. There was no statistically significant difference in the mean value in either region in both parameters. **Conclusion:** The local application of a 1% metformin solution in an animal model of guided bone regeneration with particulate bovine hydroxyapatite and hyaluronate resulted in greater bone neoformation and xenograft osseointegration than in the control group.

## 1. Introduction

Rehabilitating edentulous areas with osseointegrated implants has revolutionized dentistry, improving patient quality of life. However, bone loss and insufficiency, as features of various systemic and periodontal diseases, trauma, and tumors, remain major challenges in osseointegration [1]. Numerous techniques and subsequent modifications have been proposed to achieve adequate bone volume to support ideal three-dimensional implant placements. Autologous block grafting became the gold standard for the horizontal and/or vertical reconstruction of edentulous alveolar ridges. However, advances in biomaterials and clinical techniques have led to the incorporation of guided bone regeneration (GBR) as a potential alternative in challenging cases [2]. GBR is based on the principle of cell exclusion, where the use of membranes serves as a barrier to prevent epithelial and connective tissue from colonizing the space to be regenerated, which is usually filled with particulate grafts [3].

In addition to certain physical properties meant to maintain space throughout healing and achieve better bone neoformation, grafts in GBR should ideally have as many of the following three biological properties as possible: osteogenicity, osteoinductivity, and osteoconductivity [4]. Xenografts and, more specifically, deproteinized bovine bone minerals, are some of the most widely used types of grafts today because of the successful results they have shown in GBR procedures [5]; they are a good alternative to the gold standard but are primarily and only osteoconductive [6]. This is because, although the organic part is removed to reduce the risk of infection, the inorganic phase—mainly consisting of hydroxyapatite—and its porous architecture are preserved [7]. It is thus considered a slow reabsorption material that is still detectable after more than a decade [8]. Recently, different compounds, such as purified porcine type I collagen and sodium hyaluronate, have been added to this type of xenograft to improve its handling by increasing the cohesion between granules [9,10]. In addition to this, hyaluronic acid has been shown to achieve faster cell proliferation and vascularization and better clinical results [11].

Regarding the multiple techniques described within the subject of guided bone regeneration, many practitioners have opted to add substances or coadjuvant drugs to favor osteogenesis and cause cells that colonize the intervened area to differentiate into an osteoblastic lineage. Although much of the literature focuses on substances that can confer osteoinductivity to biomaterials, such as recombinant growth factors [12] and PRP [13], there are several molecules, such as vitamin D and metformin, that have been studied as possible adjuvants in the field of GBR owing to their biological benefits in numerous medical uses [14,15,16,17].

Metformin (dimethylbiguanide) is a synthetic guanidine derivative isolated from extracts of *Galega officinalis* [18]. Although metformin has an established role in the treatment of type 2 diabetes, recent human studies have shown some novel pleiotropic actions, ranging from various regulatory properties [19] to anti-proliferative [20] and anti-inflammatory effects [21]. Metformin has been shown to have bone-protective effects, negatively regulating RANKL in osteoclast differentiation, as well as the ability to promote osteoblast mineralization and differentiation [22]. Furthermore, metformin has been shown to positively regulate the production of OPG from osteoblasts, resulting in a decreased RANKL/OPG ratio, which promotes the induction of bone neoformation and the inhibition of bone resorption [23]; recent in vitro studies have also shown that metformin can promote osteogenic differentiation in periodontal ligament stem cells [16].

Recent studies have shown how the local application of 1% metformin to infrabony defects in periodontal patients can significantly improve clinical and radiological outcomes after scaling and root planing treatments [24,25]. Several authors have studied the effect of this drug as an adjuvant during implant osseointegration in animals [26,27], but there is no study in the literature that has evaluated the application of this drug locally as an adjuvant in GBR procedures.

Therefore, the present study aims to evaluate the effect of the local application of a 1% metformin solution on bone neoformation in the treatment of an experimental bone defect in a guided bone regeneration animal model with a particulated bovine hydroxyapatite xenograft with hyaluronate.

## 2. Materials and Methods

### 2.1. Formulation of the 1% Metformin Solution

A 1% metformin solution was prepared based on previous studies [24]. We weighed 0.128 g of metformin hydrochloride on an analytical balance and dissolved it in sterile water for injection (Laboratorios Grifols, Barcelona, Spain) using a sterile beaker on a magnetic stirrer. After this, the pH was checked, and metformin was found to be stable in aqueous solution at a pH between 4.6 and 4.9. Once the solution was obtained, it was sterilized via final filtration. For this, a sterile 10 mL syringe and a sterile hydrophilic filter with a 0.22 μm polyethersulfone (PES) membrane were used. A 10 mL sterile vial (Envases Farmacéuticos SIREP S.L., Tarragona, Spain) was used as a conditioning material.

### 2.2. Ethics Committee

This study was carried out at the animal experimentation service of the Jesús Usón Minimally Invasive Surgery Center (REGA code: ES 100370001499). This experimental study complied with the ethical and legal conditions established in R.D. 53/2013 of 1 February 2013 of the European Union for animal care and experimentation, and its protocol was approved by the Animal Experimentation Ethics Committee of the Jesús Usón Minimally Invasive Surgery Center (Cáceres, Spain) and authorized by the Animal Health Service of the Department of Agriculture, Rural Development, Population and Territory of the Regional Government of Extremadura (EXP-20220617, 24 June 2022).

### 2.3. Experimental Animals

Eight male New Zealand rabbits weighing between 3.5 and 4 kg were used. The animals were acclimatized to the conditions of the center for 21 days to avoid complications owing to the change of habitat, which could introduce bias to the study. They were kept at a temperature between 20 °C and 24 °C, with a humidity of around 55% and fifteen air changes per hour, with a 12 h light/dark cycle with intensity control, without excessive noise, and with a standard diet and water ad libitum. Each animal was housed in an individual cage with a surface area of 4670 cm^2^ and a height of 40 cm, with an elevated platform that allowed the animal to hide.

### 2.4. Anesthesia and Surgical Protocol

For surgery, specimens were premedicated intramuscularly with medetomidine at a concentration of 0.8 mg/kg (Domtor^®^, Pfizer S.A., Madrid, Spain) and ketamine at 5 mg/kg (Ketolar^®^, Pfizer S.A., Madrid, Spain). Anesthesia was induced with intravenous propofol (Diprivan^®^, Zeneca Farma S.A., Pontevedra, Spain) at a concentration of 4 mg/kg. Anesthesia was maintained via inhalation with sevofluorane (Sevorane^®^, Abbott Laboratories S.S., Madrid, Spain) at a concentration of 1.1–1.2%. To induce local anesthesia, subcutaneous articaine hydrochloride with epinephrine at 40/0.01 mg/mL (Ultracain^®^, Laboratorios Normon S.A., Madrid, Spain) infiltration was administered in the surgical area.

**Figure 1:**
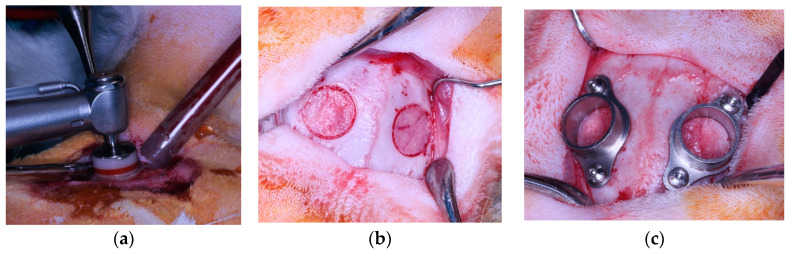
(**a**) Milling with an 8 mm diameter trephine with a stop to mark 0.5 mm; (**b**) 8 mm diameter defects without external cortex in both parietal bones; (**c**) fixing the titanium cylinders with an internal diameter of 6.9 mm and a height of 4 mm using two pins.

After preparing the working area and shaving and disinfecting the skin of the head of the rabbit to be operated on with povidone–iodine, a 5 cm long longitudinal incision was made in the medial area of the head, and a full-thickness flap was raised, exposing the parietal bones of the calvaria. To create defects in the calvaria, an 8 mm diameter trephine constantly irrigated with saline was used to delimit the circumference of the defect. The trephine was used with a stop to mark the desired depth, which was set at 0.5 mm to keep the inner cortex of the cranial bones intact. The external cortex of the working area was removed with a tungsten carbide round handpiece bur (HM141F 031, Meisinger, Neuss, Germany), resulting in two defects of 8 mm diameter and 0.5 mm depth with a medullary bone base. Using two pins (Bone Management Master-Pin-Control^®^, Meisinger, Neuss, Germany), titanium cylinders (Soadco S.L., Escaldes-Engordany, Andorra) designed specifically for this methodology and referenced in previous studies [3] were fixed. This process is shown in Figure 1.

**Figure 2:**
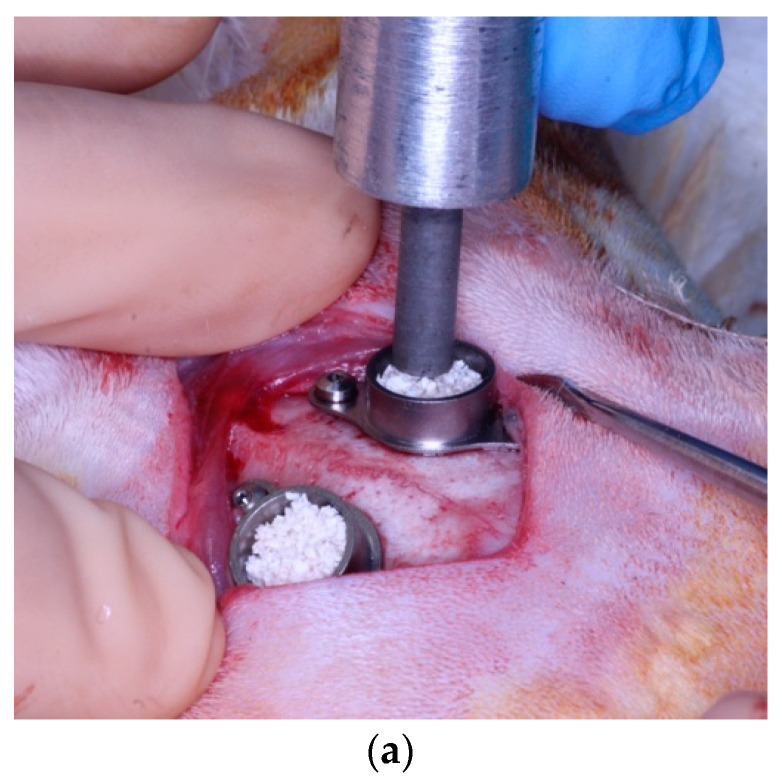

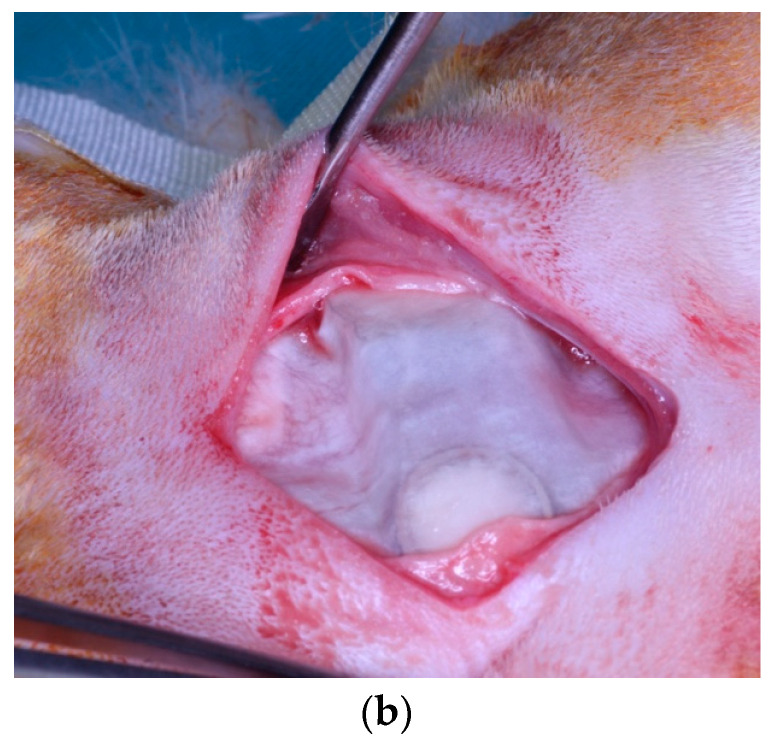
(**a**) Compaction of the biomaterial at 0.8 N and a resulting pressure of 0.7 kg/cm^2^ using a compression dynamometer. Note the absence of biomaterial in the external area of the cylinder after compaction. (**b**) Coverage of both defects with a resorbable porcine pericardium collagen membrane.

Both defects were filled with 0.105 g of particulate hydroxyapatite of bovine origin and sodium hyaluronate (Cerabone Plus^®^, Botiss Biomaterials GmbH, Zossen, Germany) with a particle size between 0.5 and 1.0 mm. The volume of graft added was verified to correspond to 112.18 mm^3^ to avoid too much variability in terms of the hyaluronate ratio and different particle sizes. We added 0.013 mL of sterile injectable saline to the weighed and flushed biomaterial samples that were to be inserted into the left parietal defects and 0.013 mL of sterile 1% metformin solution to the right parietal defects of each animal. After filling the defects, 5 standardized compressions of 0.8 N and a resulting pressure of 0.7 kg/cm^2^ were performed with a compression dynamometer [28] to control the compaction of the biomaterial (Figure 2a). The defects were completely covered with a resorbable porcine pericardial collagen membrane (Jason^®^ membrane, Botiss Biomaterials GmbH, Zossen, Germany) (Figure 2b) and sutured in layers with a 4/0 polyglycolic acid suture (Surgicryl PGA, SMI, St. Vith, Belgium).

After surgery, the animals were subcutaneously administered both antibiotic therapy (Enrofloxacin Baytril^®^, Bayer Hispania, Barcelona, Spain; 10 mg/kg every 12 h for 5 days) and analgesia (Buprenorphine Buprex^®^, RB Pharmaceuticals, Slough, UK; 0.05 mg/kg every 8 h for 3 days). They received the necessary care and were checked for symptoms or signs indicating pain, fever, and/or infection.

### 2.5. Euthanasia and Specimen Preparation

All specimens survived the healing period. At 6 weeks, the animals were euthanized via intravenous potassium chloride injection (Braun^®^, Braun Medical S.A, Barcelona, Spain. 1–2 mmol/kg) after the induction of general anesthesia.

Once the rabbit’s calvaria was exposed, the samples were cut with an oscillating saw, resecting more than 2 mm on the cranial, caudal, and distal sides, and the two samples were sectioned with the suture between the parietal bones.

The samples were fixed in 10% formaldehyde for 48 h to preserve their tissue structure and to ensure bone tissue fixation, after which they were dehydrated through immersion in solutions with increasing concentrations of ethanol. Once fully dehydrated, the samples were embedded in methyl methacrylate resin using solutions of increasing concentration and kept under vacuum conditions for 48 h to ensure that the resin penetrated the tissues properly. After this, the samples were photopolymerized in a light control unit, obtaining a transparent solid block of each sample to enable cutting.

### 2.6. Histomorphometric Analysis

The polished sample surfaces were coated with a thin layer of gold and individually observed via focused ion beam scanning electron microscopy (FIB-SEM) using a Zeiss Neon 40 Cross Beam (Zeiss, Germany) with a backscattered electron detector at 15 kV potential and 8 mm working distance to achieve a resolution down to 1.1 nm.

**Figure 3:**
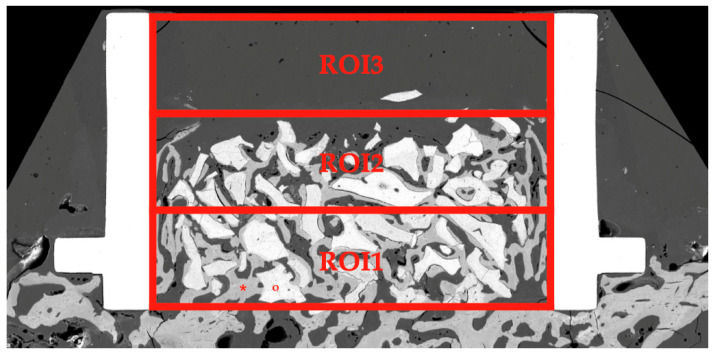
Distribution of the regions of interest (ROI1, ROI2, and ROI3) for the sample analysis after obtaining SEM images. Note the absence of biomaterial and newly formed bone in ROI3. * Indicates newly formed bone; ° indicates residual material.

The SEM evaluation was carried out by sequentially scanning polished surfaces. The images were automatically fused with the Zeiss Atlas 5 software and processed with ImageJ to calculate the percentage of new bone formation (NBF %) with respect to the total area of the region of interest and the percentage of bone–biomaterial contact (BBC %) in the different regions of interest. The internal space of the cylinder was divided horizontally into three regions of interest (ROI) of 1.3 mm height each, from proximal to distal: ROI1, ROI2, and ROI3 (Figure 3). We decided to discard ROI3 for histomorphometric analysis since the amount of biomaterial remaining in this region was negligible in all cylinders, meaning that this region was of no interest to our study. Therefore, we analyzed ROI1—more proximal to the rabbit’s calvaria—and ROI2—corresponding to the middle area of the cylinder and the region more distal to the animal’s calvaria of the two examined.

### 2.7. Statistical Analysis

The variables used in the study are described using their median, mean, and standard deviation. Likewise, the normality of all these variables was studied with the Anderson and Darling test [29]. Similarly, homoscedasticity was tested with the Bonett [30] and Levene [31] tests.

For the parametric variables, the *t*-test was used (paired or unpaired, depending on the circumstances) to detect differences between the means of the different groups [32]. For the non-parametric variables, the Wilcoxon [33] test was used as a paired test, and the Kruskal–Wallis [34] test was used as an unpaired test.

## 3. Results

**Figure 4:**
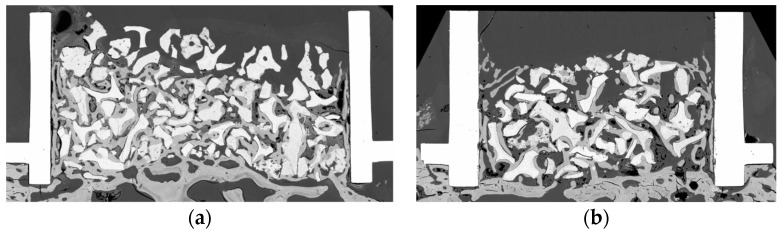
(**a**) Focused ion beam scanning electron microscopy image of one of the cases treated with 1% metformin. Note the absence of biomaterial in much of the third most distal to the shell of the interior of the cylinder. (**b**) Focused ion beam scanning electron microscopy image of one of the control cases. Note the absence of biomaterial in much of the third most distal to the shell of the interior of the cylinder.

No complications were recorded during the cylinder fixation surgery and subsequent placement of the membrane and suture, and all the animals passed the healing phase correctly, without pain, fever, or any other signs of infection. The histomorphometric analysis showed bone neoformation to varying degrees and in all cases, with correct xenograft integration with the native parietal bone, as well as bone formation on the inner surface of the cylinder (Figure 4). The descriptive statistics of this analysis are shown in Table 1.

### 3.1. New Bone Formation

**Figure 5:**
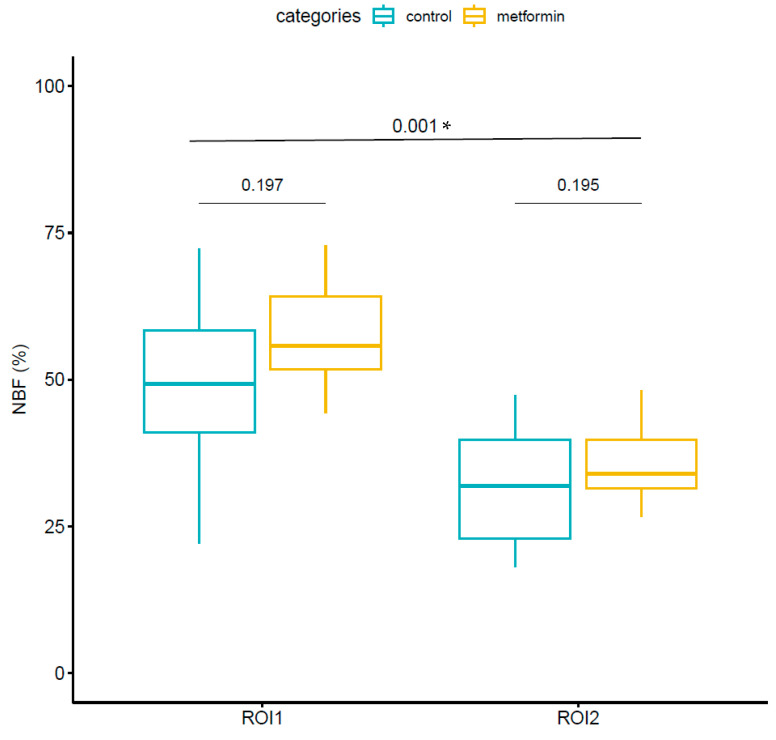
Histomorphometric measurements of new bone formation percentage in each region of interest, represented by box plots. The median, percentiles, and maximum and minimum values of each group are shown. At the top, we can see the *p*-values obtained after applying the paired *t*-test comparing cases treated with metformin and controls within each region of interest and comparing the two regions of interest overall; “*” indicates statistically significant differences.

In region of interest 1 (ROI1), the region closest to the animal’s calvaria, a percentage of new bone formation (NBF %) of 57.74 ± 9.83% was observed in the metformin samples, and 46.75 ± 15.80% was observed in the control samples. After applying a paired *t*-test, values of T = 1.43 and *p* = 0.197 were obtained which showed that there were no statistically significant differences in their mean values when comparing the metformin cases with the control cases. In ROI2, the region most distal to the animal’s calvaria, a new bone formation percentage of 36.06 ± 7.42% was observed in the metformin samples, and 28.64 ± 10.80% was observed in the control samples. After applying a paired *t*-test, values of T = 1.43 and *p* = 0.195 showed that there was no statistically significant difference in the mean value when comparing the metformin cases with the control cases. ROI1 showed higher values than ROI2 in terms of new bone formation percentage (52.2 ± 13.9% and 32.35 ± 9.74%, respectively). After applying a paired *t*-test, values of T = 4.68 and *p* = 0.001 were obtained, which allowed us to affirm that there were statistically significant differences between the two regions. These results are shown in Figure 5.

### 3.2. Xenograft Integration

**Figure 6:**
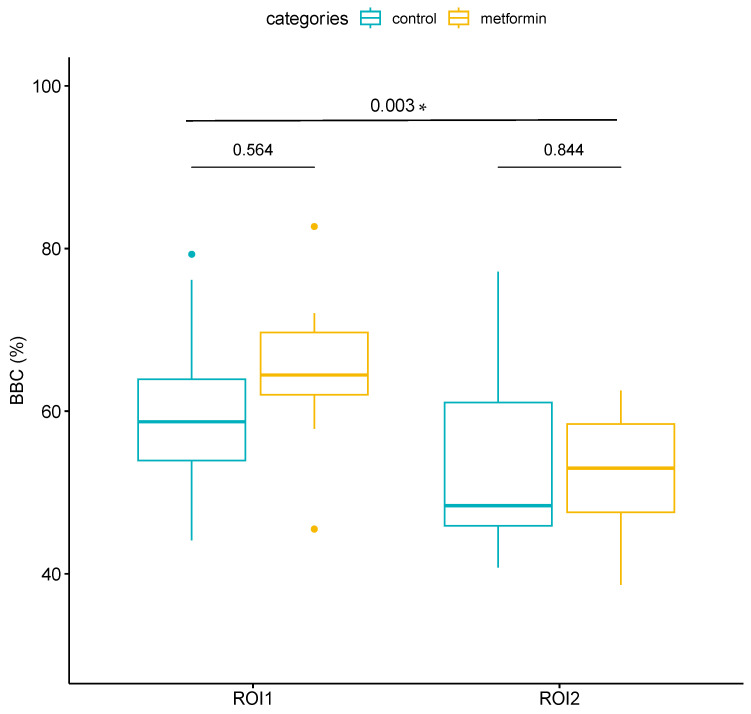
Histomorphometric measurements of the bone–biomaterial contact percentage (BBC %) in each region of interest, represented by box plots. The median, percentiles, and maximum and minimum values of each group are shown. At the top, we can see the *p*-values obtained after applying the paired *t*-test comparing cases treated with metformin and controls within each region of interest and comparing the two regions of interest overall; “*” indicates statistically significant differences.

In ROI1, an average bone-to-biomaterial contact percentage (BBC %) of 64.93 ± 10.77% was observed in the samples with metformin, and 61.26 ± 12.37% was observed in the control samples. After applying a paired *t*-test, values of T = 0.61 and *p* = 0.564 were obtained, which confirmed that there were no statistically significant differences in their mean value when comparing the metformin cases with the control cases. In ROI2, an average bone–biomaterial contact percentage of 52.31 ± 7.92% was observed in the metformin samples, and 51.35 ± 8.63% was observed in the control samples. After applying a paired *t*-test, values of T = −0.20 and *p* = 0.844 were obtained, evidencing that there were no statistically significant differences in their mean values when comparing the metformin cases with the control cases. ROI1 presented higher values than ROI2 in the average bone–biomaterial contact percentage (63.1 ± 11.4% and 51.83 ± 8.02%, respectively). After applying a paired *t*-test, values of T = 3.24 and *p* = 0.003 were obtained, making it possible to affirm that there were statistically significant differences between the two regions. These results are shown in Figure 6.

## 4. Discussion

Osteoconduction is defined as the ability to provide a microscopic and macroscopic passive scaffold that facilitates bone formation and growth by providing a porous structure through which cells and blood vessels can migrate and develop, leading to the formation of new bone tissue [4]. Because of their high availability, biosafety, and low cost, xenografts are one of the most widely used biomaterials in this type of procedure, but although they have good osteoconductivity [6], they lack the other two qualities. This is why adding different growth factors or drugs to these biomaterials has been postulated as a promising strategy for achieving better results [13,35,36,37,38,39,40]. One of these adjuvants is recombinant human bone morphogenetic protein-2 and rhBMP-2, and its use has been widely documented in the literature as an adjuvant for deproteinized bovine-derived bone mineral. Even so, despite showing satisfactory results even in long follow-up periods, there are concerns about the safety of rhBMP-2 regarding its potential protumorigenicity, which is why many clinicians prefer other adjuvants [36]. Another adjuvant that provides growth factors is plasma rich in growth factors (PRGF), which has demonstrated multiple benefits in terms of tissue regeneration. Despite this, obtaining it requires a process that, while minimally invasive, requires specialized devices and qualified personnel, as venous blood has to be drawn from the patient [13]. Metformin is a safe drug with a clear capacity to activate osteoblasts and inhibit osteoclasts, according to numerous in vitro studies [15,22,41], so it has been proposed as an alternative non-invasive and safe co-adjuvant.

In all cases, the present study shows bone tissue regeneration beyond the bony framework of the rabbit’s calvaria at 6 weeks of healing. The bone neoformation percentage was higher in the metformin-treated group than in the control group, although there were no statistically significant differences between the two groups. In both the metformin-treated defects and controls, the amount of newly formed bone was significantly higher in the proximal third of regeneration—with approximately 58% of bone tissue in the metformin samples and 47% in the control samples—than in the middle third—showing percentages of approximately 36% and 29% newly formed bone tissue, respectively. The osseointegration of the deproteinized bovine xenograft was adequate in both groups, and no statistically significant differences between them were found, with approximate bone–biomaterial contact rates of 52–65% achieved in the metformin group and 51–61% in the control group.

These results are in line with those obtained previously by Viteri-Agustín et al. [3], who studied the effect on bone regeneration in the vertical direction by applying different biomaterial compression forces in a model of a critical defect in a rabbit calvaria. Despite not observing statistically significant differences between the different compression forces, they did obtain significantly higher percentages of bone neoformation in the regions closest to the calvaria in both groups, albeit with lower values (29.0 ± 8.8% in the cases of lower compression forces and 27.6 ± 8.2% in the cases of higher compression forces) than the cases in our study. When performing the histomorphometric analysis, the zones of interest were established by dividing the entire inner zone of the cylinder into internal and external zones. In our case, we interpreted the results by dividing the sample into three thirds and discarding the external one, so logically, our internal region of interest showed higher newly formed bone values, as the sample was from a smaller region and closer to the native bone. Even so, the values we obtained in terms of bone tissue formation were much higher than those obtained in studies on rabbit calvarias using similar methodologies with deproteinized mineral xenografts of bovine origin, where bone neoformation percentages of between 11.7 and 13.8% were obtained [42,43].

As mentioned above, one of the most important characteristics of xenografts for guided bone regeneration is their osteoconductivity. Therefore, regeneration with this type of biomaterial requires it to become embedded in the newly formed bone. This is why the greater the osteoconductive capacity, the greater the contact between the newly formed bone tissue and the graft, and vice versa. We can see how the integration of the graft is another important parameter when assessing variations in bone regeneration. For this reason, many authors have analyzed this parameter to determine whether the hypotheses of their work were fulfilled. This is the case of the work published by Zhou et al., in which one of the parameters they analyzed was the degree of contact between the particles and bone when assessing the effect of particle size on regeneration with inorganic bone [44]. If we look at the results obtained in the small-particle-size group, we can observe percentages of contact between particles and bone of 39.2 ± 6.7% at 4 weeks and 56.8 ± 7.9% at 10 weeks, values lower than the average of ROI1 in our study (63.1 ± 11.4%) and similar to that of ROI2 (51.83 ± 8.02%). The authors suggest that, in the small-particle group, the newly formed bone formed a greater number of bridges and connections, in contrast to the large-particle group, where the bone tissue was confined to the perimeter of the particles with fewer connections.

Few authors have studied the effect of metformin administration as an adjuvant in oral regeneration processes, and the vast majority of their studies have been directed at the regeneration of periodontal defects [25,45,46,47]. The study most similar to ours was published by Mitra et al., wherein they analyzed the effect of adding 1% metformin gel to the intraosseous treatment of chronic periodontal patients with demineralized and freeze-dried bone allografts (DFDBAs) [40]. These authors found better probing values, shallower defect depths, and greater bone height in the group that underwent regeneration with DFDBAs mixed with 1% metformin compared with a control group treated with DFDBA regeneration alone. However, as in our study, although improvements were observed in all areas, the differences between the groups were not statistically significant. As this was a human study, histological studies could not be carried out, and all the values relating to bone neoformation were based on clinical and radiological parameters; therefore, although the results were of greater relevance, we cannot confirm the beneficial effect of the drug on bone neoformation.

There are a few limitations in this study. The first is the model used. Although the experimental rabbit calvaria model has many advantages, it is not representative of a possible guided bone regeneration situation in humans, both because of its macro- and microstructures and because it has a higher rate of bone remodeling [48]. This means that the results cannot be extrapolated and that the experimental model in rabbits is a first step toward experiments in larger animal models or with bone tissues more similar to those of humans. Another limitation is the small sample size, with a single healing period and a single concentration of metformin tested. However, we consider this study to be a first step for future experimental studies with larger sample sizes and different healing times and/or studies with larger experimental animals that would enable a greater number of defects in the same specimen. The third limitation is that although the regeneration model used in this study allowed us to control the volume and pressure applied during graft placement, it did not represent a common clinical situation. In addition, the regenerated area within the cylinder in the rabbit’s calvaria does not receive the mechanical stimuli of the maxillary or mandibular areas, as it lacks the movements generated by the masticatory system [49,50]. Finally, the vertical model created within the cylinder had only one osteogenic front and was not contained, resulting in significant differences in bone neoformation in the proximal areas compared with the distal areas.

Bearing these limitations in mind, the present study opens a research avenue for future experimental studies assessing the effects of metformin on bone neoformation when applied at different concentrations; its effect on longer healing times; and even its possible use as a co-adjuvant for different biomaterials.

## 5. Conclusions

Our results show that the local application of a 1% metformin solution in an animal model of guided bone regeneration with particulate bovine hydroxyapatite and hyaluronate resulted in greater bone neoformation and xenograft osseointegration than in a control group. Regions closer to the calvaria had significantly higher percentages of both newly formed bone tissue and bone–biomaterial contact. Metformin appears to have a promising use as an adjuvant in guided bone regeneration. However, further studies with larger sample sizes and longer-term studies are needed before clinical recommendations can be established.

## Figures and Tables

**Table 1:** Descriptive statistics of the histomorphometric analysis.

		Metformin	Control	Total
NBF %	ROI1	57.745 ± 9.832 [55.785]	46.748 ± 15.796 [49.290]	52.240 ± 13.917 [52.773]
	ROI2	36.058 ± 7.420 [33.992]	28.646 ± 10.803 [25.593]	32.352 ± 9.737 [32.192]
BBC %	ROI1	64.93 ± 10.77 [64.47]	61.26 ± 12.37 [58.7]	63.09 ± 11.36 [63.91]
	ROI2	52.31 ± 7.92 [52.99]	51.35 ± 8.63 [48.39]	51.83 ± 8.02 [50.36]

BBC %: percentage of bone–biomaterial contact; NBF %: percentage of new bone formation with respect to the total area of the region of interest. Values are expressed as mean ± SD [median].

## Data Availability

The raw data supporting the conclusions of this article will be made available by the authors on request.
